# Algae colonisation of brick pavement at the University of Venda: A potential slippery hazard

**DOI:** 10.4102/jamba.v11i2.689

**Published:** 2019-07-04

**Authors:** Thabelo R. Munyai, Thantaswa Sonqishe, Jabulani R. Gumbo

**Affiliations:** 1 Department of Ecology and Resource Management, University of Venda, Thohoyandou, South Africa; 2 Department of Hydrology and Water Resource Management, University of Venda, Thohoyandou, South Africa

**Keywords:** Green Algae, Biodeterioration of Bricks, Heavy Metals, Nutrients, Mucilage, Solar Radiation

## Abstract

A brick pavement, tramped by humans, is exposed to atmospheric elements, thus allowing cyanobacteria and algae to colonise. In this article, we report on the factors that contribute to the slipperiness of a brick pavement at the University of Venda in the Limpopo province of the South Africa. Samples were collected from brick surfaces either colonised by green algae (treated) or not (control). The samples were acid-digested and analysed for metals by Inductively Coupled Plasma Mass Spectrometry (ICP MS) in parts per billion (ppb). The treated bricks, with green algae, had average high metal contents (ppb): Al 9456.02, Ti 731.23, V 46.44, Cr 78.85, Mn 862.93, Fe 16295.18, Co 23.57, Ni 59.36, Cu 66.31, Zn 160.57, As 7.92, Se 10.45, Mo 6.74, Cd 5.19, Sn 4.65, Sb 2.31 and Pb 19.51. In contrast, control bricks had a low average of metal content (ppb) as follows: Al 2.99, Ti 0.28, V 4.04, Cr 1.42, Mn 4.29, Fe 20.89, Co 0.36, Ni 2.74, Cu 5.64, Zn 4.21, As 0.56, Se <3.00, Mo 0.88, Cd 0.01, Sn 1.05, Sb 0.04 and Pb 0.04. Other factors that promote algae colonisation include high solar radiation, neutral pH, nutrients, low electrical conductivity and total dissolved solids. The algae colonisation of brick pavement results in an unaesthetic sighting and a slippery surface that is hazardous to humans.

## Introduction

Assessing and reducing the likelihood of disaster occurring is part of disaster planning and disaster reduction strategy. A pavement is used daily by people commuting to and from work. The pavement is exposed to atmospheric elements such as rain, sunshine and inhabitation of cyanobacteria, thus allowing it to be slippery; this may result in injury to persons walking on the pavement. Thus, bacteria, cyanobacteria and algae are known to colonise stone monuments, statues, pavements, and historic places and buildings on a daily basis. The factors that contribute to the colonisation of these agents include moisture and nutrient availability, favourable pH, essential and trace metal availability and favourable solar radiation. Because of these favourable conditions, cyanobacteria and Chlorophyta (green algae) are the early inhabitants (Cecchi et al. 2000; Crispim & Gaylarde 2005; Ortega-Calvo et al. 1991; Tomaselli et al. 2000a). Cyanophyta and Chlorophyta are photoautotrophs that use atmospheric carbon dioxide in the presence of sunlight captured in chlorophyll *a*, nutrients and moisture to synthesise and produce carbohydrates, oxygen and adenosine triphosphate (Barsanti et al. 2008). The photosynthetic pigments chlorophyll *a,* phycobilins and carotenoids use water (H_2_O) as electron donor and release O_2_ exclusively to Cyanophyta (blue green algae), and pigments chlorophyll *a, b* and carotenoids use both photosystems I and II and are exclusive to Chlorophyta (green algae) (Lembi & Waaland 1988; Tomaselli, Tiano & Lamenti 2000b).

The presence of moisture and nutrients, nitrates and phosphates triggers a proliferation of cyanobacteria growth. Fried, Mackie and Nothwehr (2012) suggested that phosphate and nitrate levels lower than 0.05 mg/L can significantly hinder algal growth. However, some cyanobacteria species such as *Anabaena, Cylindrospermopsis* and *Lyngbya* are able to harvest atmospheric nitrogen and make it available for growth purpose (Paerl 2008). Thus, the low availability of phosphorus slows the growth of cyanobacteria.

The availability of trace and essential metals is important in the metabolism and photosynthesis of cyanobacteria, thus contributing to their growth and propagation. Huertas et al. (2014) have shown that iron is a co-factor in all three photosynthetic electron transport systems, manganese is part of photosystem II, magnesium coordinates the elements in the chlorophyll and zinc is part of the enzyme carbonic anhydrase. Zinc is a familiar essential micronutrient for normal growth of algae (Chaoui et al. 1997) and is also known to transform carbohydrates and parts of enzyme systems that regulates the plants growth; it also regulates the use of sugar. Vassiliev et al. (1995) described that iron shortage causes a significant reduction in the abundance of light-harvesting complex II (LHCII) proteins; hence, proteins containing iron are essential for photosynthetic and respiratory electron transport and are directly involved in nitrate and nitrite reduction, N_2_ fixation, chlorophyll synthesis and a number of other biosynthetic or degradative reactions. In algae, iron-containing catalysts also play an important indirect role in cellular metabolism by regulating enzyme activity (Scheibe 1991); it is essential for the biosynthesis of chlorophyll molecules (Yu, Chen & Zhang 2015). Manganese is used in plants and algae as a major contributor and as a co-enzyme involved in photosynthesis, respiration and nitrogen assimilation (Kochian, Hoekenga & Piñeros 2004). Copper is another cofactor that is essential for the management of oxidative stress response and in co-enzymes such as cytochrome c oxidases because of its ability to switch from Cu^2+^ and Cu^+^ (Huertas et al. 2014). However, at high levels of copper, it is used as an algaecide, that is, killing of cyanobacteria (Gumbo & Cloete 2013).

Cyanobacteria are known to thrive in alkaline pH. According to Ascasoa, Wierzchosb and Castello (1998), cyanobacteria complexes with carbonate ions (in limestone building) with a pH value of >8.3 result in bicarbonate ions that are acted upon by carbonic anhydrase and converted into CO_2_ and OH^–^. The OH^–^ ions are released and concentrated around the cells, producing a localized microenvironment of high pH. However, Van der Oost et al. (1989) showed that some cyanobacteria species were able to carry out mixed acid fermentation, which resulted in corrosion of stone building. Waterbury (1989) suggested that it is the associated heterotrophic bacteria that lead to acid decay of calcareous materials, in the presence of cyanobacteria.

Cyanophyta (blue green algae) and Chlorophyta (green algae) have been found growing in different habitants and producing mucilage, a slippery substance. In Turkey, a study by Selvi and Altuner (2007) found that Ballica cave walls and floors that were dominated by the Cyanophyta group had become slippery because of the sheaths with mucilage and gelatinous texture of the blue-green algae. The factors that promote the growth of cyanobacteria were moisture, low light conditions and limestone structure that provide the essential nutritional elements.

The Cyanophyta group – *Nostoc* spp., *Oscillatoria* spp., *Cosmarium* spp., *Cylindrocystis* spp., *Dactylothece* spp., *Klebsormidium* spp., *Mesotaenium* spp. and *Ourococcus* spp. – were found to produce copious amounts of mucilage in US golf greens and four sites in northern England (Baldwin & Whitton 1992). The factors that favoured the growth of these organisms included excess surface water flowing across the golf turf, sunlight, cold conditions, soil compaction (which reduces water infiltration), low soil fertility with ammonium sulphate and soil pH. In acidic soils, the cyanobacteria were able to grow well producing mucilage compared to alkaline soil pH (Baldwin & Whitton 1992). The mucilage is composed of sulphated polygalactans that are responsible for the slippery characteristics of building floors and pavements after rain period (Nelson & Cox 2013).

Smith and Olson (2007) also found that the Cyanophyta group was dominate on walls and floors of Mammoth Cave National Park, south central Kentucky, USA, where white fluorescent, incandescent flood lights and later 500 W halogen (yellow Light-emitting Diode [LED] lights) were installed. The cyanobacteria growth was enhanced by the presence of moist air and sufficient lighting, which enable the organisms to carry out photosynthesis. In another study by Olson (2006) on the lighting systems within the Mammoth Cave National Park, yellow LED lights that emitted in the 595 nm region were installed in one section of the cave. In this section, it was noticed that there was no cyanobacteria growth in comparison to the other areas where the white fluorescent and incandescent flood lights were used. The use of LED lights to manage the presence of cyanobacteria and their slippery mucilage looks promising as compared to the previous use of mechanical scrapers, bleach and algaecides and steam. However, cyanobacteria species are cosmopolitan and may adapt to low light composition by increasing specific phycobiliproteins that will adsorb at the specific wavelengths (Smith & Olson 2007).

**FIGURE 1:**
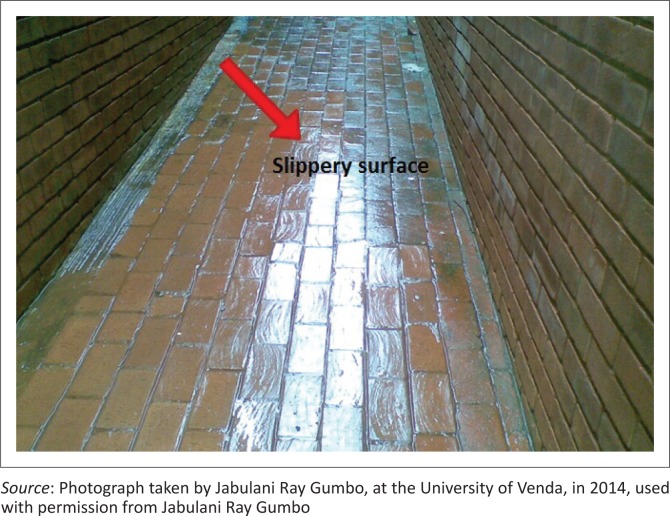
Algae growing on a building’s wall and pavement.

The presence of algae on the pavement causes the brick surface to be slippery (Figure 1). This is hazardous because a person can easily slip and get injured. Secondly, the presence of the green colour makes the pavement unsightly to look at (the aesthetic appearance of the green colour is not attractive).

The purpose of this study was to assess the role of cyanobacteria (cyanophyta and chlorophyta), collectively termed as algae, in the biodeterioration of buildings and paving bricks. Some bricks or buildings seem not to have these algae that deteriorate most infrastructure; therefore, the main idea of this research was to find the factors that promote the growth of algae on certain bricks and their absence in other bricks. The specific research objectives were to determine the chemical and physical quality of rainwater (pH, electrical conductivity [EC], total dissolved solids [TDS], nitrates and phosphates) and the metallic content of the bricks (affected by algae and the control).

## Materials and methods

### Sample collection and preparation

**FIGURE 2:**
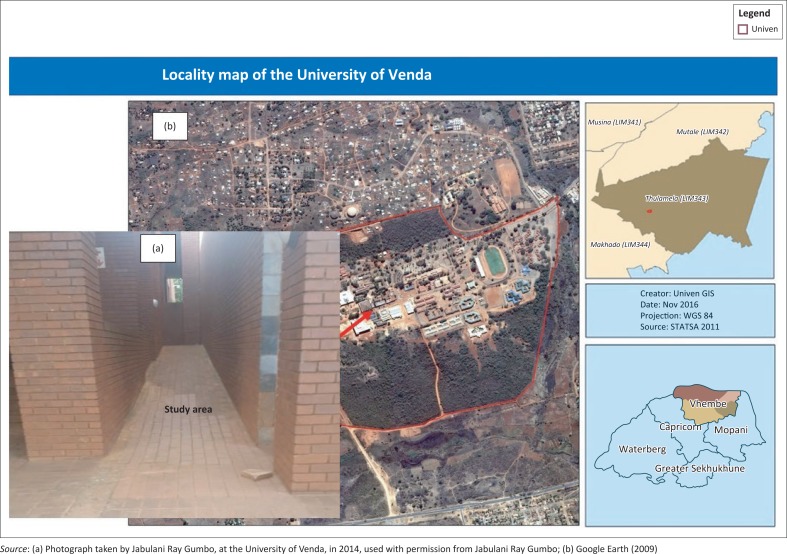
The location of the pavement at the University of Venda building.

The rain water samples were collected on rainy days at least twice in January 2014 (Figure 2). The physical tests – pH, EC, light intensity, turbidity and temperature – were carried out on-site to avoid change in sample condition. The physical tests were performed using a Crison Multimeter that was calibrated for quality control and all tests were conducted in triplicate.

Six paving bricks were scrapped using chisel and hammer to obtain 2 g of brick material (Figure 2). The brick material was then transferred into six separate 50 mL plastic bottles for storage. A mortar and pestle was used to grind the brick material into a fine powder; the grinding utensils were washed thoroughly and rinsed with deionised water to avoid any contamination. The fine powder was transferred into separate petri dishes dried in an oven at 50 °C for 24 h to remove any moisture and then was cooled in a desiccator for 24 h.

An amount of 100 mg of solid samples was weighed on an analytical balance and transferred into 250 mL conical flasks where 30 mL of concentrated nitric acid was added to each flask for digestion. The samples in reaction flasks were heated on a hot plate for 48 min to aid the digestion process while constantly swirling the flask. The remaining contents of the flask were transferred into a 100 mL volumetric flask filled to mark with deionized water and mixed thoroughly for homogeneity. The mixture was then filtered through a 0.45 µm membrane filter and transferred to 100 mL plastic bottles for storage. The samples were stored in a refrigerator in preparation to be sent to an independent laboratory in Pretoria for ICP-MS trace metal analysis. The nitrates and phosphates in rain water samples, were analysed using test kits on the Spectroquant Pharo 100 equipment in duplicate samples.

### Nitrate analysis

A nitrate test was performed using Spectroquant Pharo 100 and the NO_3-_N method, and the samples were analysed in duplicate. The rain and surface running water samples were filtered using a 0.45 µm membrane filter. An amount of 0.50 mL of the filtered sample was transferred into a self-test bottle and 10 mL of reagent NO_3_-1K was added to the solution and left to react for 10 min. Then the solutions were transferred to the Spectroquant Pharo 100 for nitrate analysis to determine its concentration levels.

### Phosphate analysis

A Phosphate test was conducted using Spectroquant Pharo 100 and the PO_4_-P method and the samples were analysed in duplicate. The samples of surface running and rain water were filtered using a 0.45 µm membrane filter. An amount of 10 mL of sample water was poured into a test tube for each sample, then five drops of reagent P-2K were added, followed by one dose of reagent P-3K, and the samples were left to react for 5 min. Then the solutions were transferred to the Spectroquant Pharo 100 for phosphate analysis to determine its concentration levels.

### Data analysis

The variation in metal composition from the six bricks was tested using one-factor analysis of variance (ANOVA), with the level of significance set at *p* < 0.05. Standard deviation was used for analysing variance in metal compositions, phosphates, nitrates, pH, EC, TDS and light. The metal content was expressed on dry weight basis in milligrams per litre (mg/L).

### Ethical considerations

No animal and or human participants were involved in the study and hence there was no application for permission from then University of Venda ethics committee.

## Results and discussions

### Algae colonisation of pavement bricks

**FIGURE 3:**
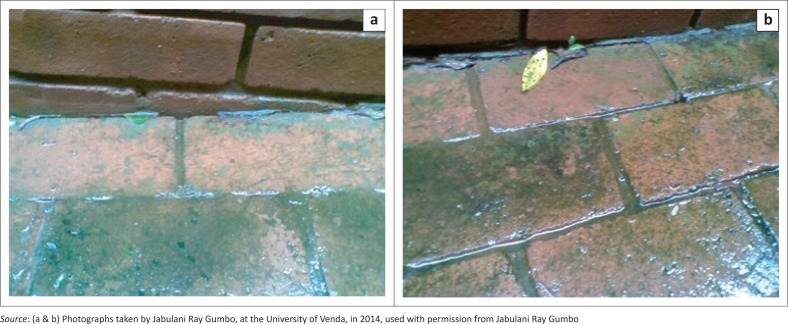
(a & b) presence of algae on the pavement bricks during a rain event.

**FIGURE 4:**
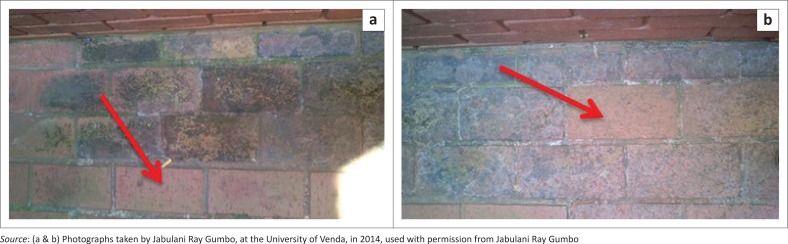
Some (a) pavement bricks with no presence of algae and some (b) pavement bricks with the presence of algae (pointed by red arrow).

**FIGURE 5:**
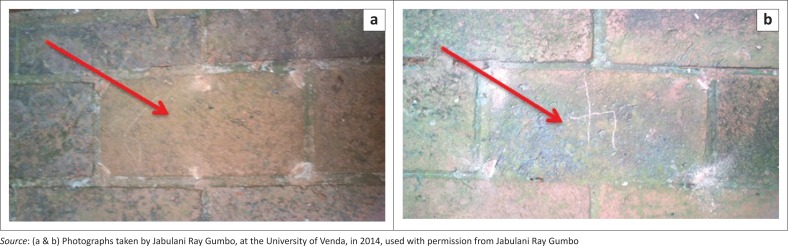
(a) Pavement brick with minimal presence of algae and (b) pavement brick with high levels of algae

On visual inspection of the pavement bricks, a green colouration was observed during a rain period (Figure 3). Some of the pavement bricks showed minimal or no green colouring (Figures 4 and 5). After the rain period, the green colouration turned black. The visual appearance of green colouration of this slippery substance was attributed to algae. The change of green to black colour was probably because of the sunlight bleaching the algae and/or the algae were photosensitive and adopted a defensive strategy to survive the sunlight radiation. These black slimes were also observed by Baldwin and Whitton (1992) on golf turf grass where cyanobacteria were growing. The mucilage of the algae was attributed to the slippery characteristics, which was because of sulphated polygalactans, a photolight protector (Nelson & Cox 2013).

The colour change may be attributed to high light intensity that turns green algae to black, and they become less slippery when they were black because there will be less water in surrounding environment. The effect of slippery pavement was also noticed by Quadri and Sidek (2014) who attributed this to the presence and growth of algae.

### Physical–chemical characteristics at study site

The pH was slightly alkaline for both rain water and surface running water (Table 1). According to Tucker and D’Abramo (2008), this alkaline pH is one of the contributing factors to the growth of algae. According to Karius and Hamer (2001), the alkaline pH may also contribute to the leaching of heavy metals from the pavement bricks at trace levels. In the study of the Tengger desert, China, Hu et al. (2002) found that the algae were able to stabilise and aggregate fine sand together in alkaline pH.

**TABLE 1:** The physical–chemical characteristic of the water samples collected on 27 January 2014.

Water samples	pH	EC (µS/cm)	TDS (mg/L)	Nitrate (mg/L)	Phosphate (mg/L)
Rain water	7.12±0.03	3.77±0.15	2.24±0.10	<1.0	2.1±0.2
Surface running water^a^	7.18±0.089	3.55±0.69	2.26±0.43	10.1±0.5	1.4±0.0

EC, electrical conductivity; TDS, total dissolved solids.

a , Water that was flowing on the ground covering the pavement bricks after a rain event.

The electrical conductivity and total dissolved solids were slightly high for both water samples (Table 1). The present levels of EC and TDS can also promote the growth of algae (Ela 2007). Although the rain water had low levels of nitrates and phosphates, the surface running water had ample levels of nutrients to stimulate the growth of algae (Table 1). Thus, the presence of alkaline pH and nutrients in the surface running water would promote the growth of algae (Fried et al. 2012).

The mean solar radiation was measured as light intensity (lux) and converted to photosynthesis photon flux density and recorded as 19.7±1.2 µ mol m^–2^s^–1^ (Table 2). The solar radiation reading is similar to the study of Barberousse et al. (2007) who showed that photosynthesis photon flux density of 15 µ mol m^–2^s^–1^ would promote photosynthesis and the growth of algae in an indoor experiment. Smith and Olson (2007) also found that the Cyanophyta group was dominant on walls and floors of Mammoth Cave National Park, south central Kentucky state, USA, where the light intensity was high because of artificial lighting. Thus, the availability of moisture, nutrients, alkaline and favourable solar radiation can promote the propagation of algae on brick pavement.

**TABLE 2:** The solar radiation at the study site on 27 January 2014.

Replicates	Light intensity (lux)	Photosynthesis photon flux density (µ mol m^–2^s^–1^)
1	1178	19.7
2	1245	20.9
3	1098	18.4
Average ±SD	1174±74	19.7±1.2

SD, standard deviation.

### Metal content of pavement bricks

The study results showed that heavy metal concentrations on pavement bricks were variable among the pavement bricks (Table 3). The pavement bricks where there was algae indicated low metal content (control) than pavement bricks (treated) with pronounced algae presence; this was confirmed by the significant difference between the two samples (*p* < 0.05). According to Karius and Hamer (2001), the alkaline pH may contribute to leaching of heavy metals from the pavement bricks at trace level. This means that the algae may be able to obtain the essential metals from these bricks and utilise them for different biological processes. In this study, no hardness tests were conducted on the pavement bricks to evaluate the leaching of heavy metals except that the brick pieces were acid-digested and then analysed for major and trace metals. Here, we report on a few metals – Zn, Fe, Cu, Mn, Ni and Co – that are essential to algae growth, photosynthesis and propagation.

**TABLE 3:** The composition of metals in the pavement bricks and the presence of algae.

Metals	No algae (mg/L)^a^	Algae1 (mg/L)^b^	Algae2 (mg/L)^b^	Algae3 (mg/L)^b^	Algae4 (mg/L)^b^
Al	2.99±0.04	2748.17±35.60	11 460.00±148.45	16 740.00±216.84	8669.00±112.29
Ti	0.28±0.02	137.92±8.57	975.30±60.61	1252.80±77.86	577.85±35.91
V	4.04±0.34	17.41±1.49	60.48±5.16	78.16±6.67	42.03±3.59
Cr	1.42±0.15	26.01±2.83	96.26±10.46	146.55±15.93	63.99±6.96
Mn	4.29±0.28	195.84±12.83	1002.50±65.67	1865.77±122.22	526.28±34.47
Fe	20.89±1.83	5729.69±503.15	22 380.00±1965.31	28 640.00± 2515.03	12 500.00±1097.69
Co	0.36±0.03	5.65±0.46	26.77±2.19	50.97±4.17	14.62±1.20
Ni	2.74±0.19	21.61±1.47	79.32±5.41	108.09±7.37	42.89±2.92
Cu	5.64±0.38	39.87±2.66	98.41±6.55	96.24±6.41	53.48±3.56
Zn	4.21±0.39	104.87±9.66	217.04±19.99	241.89±22.28	138.03±12.72
As	0.56±0.02	2.48±0.09	8.17±0.30	14.06±0.51	6.47±0.23
Se	3.00±0.27	3.00±0.27	11.63±1.06	24.16±2.21	3.00±0.27
Mo	0.88±0.07	3.29±0.27	11.29±0.92	6.09±0.50	5.61±0.46
Cd	0.01±0.00	0.14±0.01	0.55±0.02	0.80±0.04	18.71±0.84
Sn	1.05±0.00	1.42±0.01	5.11±0.02	5.54±0.02	5.23±0.02
Pb	0.04±0.00	6.83±0.02	23.11±0.07	32.82±0.10	17.87±0.05

Al, aluminium; Ti, titanium; Cr, chromium; Mn, manganese; Fe, iron; Co, cobalt; Ni, nickel; Cu, copper; Zn, zinc; As, arsenic; Se, selenium; Mo, molybdenum; Cd, cadmium; Sn, tin; Pb, lead.

a , No algae refers to no presence of algae on pavement brick (control).

b , Algae 1–4 refer to heavy presence of algae on pavement bricks (treated).

The pavement bricks with a heavy presence of algae showed that the bricks had a Zn content in the range of 104.8 mg/L – 241.9 mg/L on a dry weight basis (Table 3), whereas the pavement bricks with no algae or minimal algae had a Zn concentration of about 4.2 mg/L on a dry weight basis. The Zn metal is an essential micronutrient for normal growth of algae (Chaoui et al. 1997) and is also known to transform carbohydrates and part of enzymes system that regulates plants growth. The pavement bricks with a heavy presence of algae showed that the samples from these bricks had an Fe content in the range of 5729.7 mg/L – 28 640.0 mg/L on a dry weight basis (Table 3), whereas the pavement bricks with no algae or minimal algae had an Fe concentration of about 20.9 mg/L on a dry weight basis. The presence of Fe is essential for the algae growth as Fe contributes to the biosynthesis of chlorophyll which imparts the green colour (Morales, Abadía & Abadía 1990). Iron is central to algae photosynthetic electron transport systems working as a co-factor, with manganese as a part of photosystem II, while magnesium coordinates elements in the chlorophyll and zinc is part of the enzyme carbonic anhydrase (Huertas et al. 2014). Thus, the heavy presence of Fe in the brick content would certainly lead to algae colonisation of the brick pavements.

The range of Cu content in the pavement bricks with heavy presence of algae was 39.9 mg/L – 98.4 mg/L on a dry weight basis, whereas the bricks with no or minimal algae had a Cu concentration of about 5.6 mg/L. Cu is an essential micronutrient for plants and microbes because of the components of numerous proteins and enzymes involved in a variety of metabolic pathway (Morelli & Scarano 2004). The presence of Cu would assist the algae in the management of oxidative stress and as co-enzymes such as cytochrome c oxidases because of its ability to switch from Cu^2+^ and Cu^+^ (Huertas et al. 2014). This added advantage would confer a greater colonising role on algae for being able to manage and adapt survival strategies.

The range of Mn content in the pavement bricks with a heavy presence of algae was 195.8 mg/L – 1865.8 mg/L on a dry weight basis, whereas the bricks with no or minimal algae had an Mn concentration of about 4.3 mg/L. Manganese is an essential micronutrient and functions together with enzyme systems involved in the breakdown of carbohydrates and nitrogen metabolism, as well as assisting in the biosynthesis of chlorophyll and co-enzymes in photosynthesis processes, involving photosystem II (PSII), which provides the necessary electrons for photosynthesis (Buchanan & Jones 2000). Thus, the presence of Mn would confer on algae the ability to carry out photosynthesis process and respiration and nitrogen assimilation (Kochian et al. 2004).

Huertas et al. (2014) have also shown that Ni and Co in trace level are essential for cellular physiology of the cyanobacteria. Ni is found in two metalloproteins, namely, the methyl-coenzyme M reductase and enzymes containing the hydrogenase. Co is an important element in the uptake of vitamin B12. The Ni-coenzymes are part of the uptake of nitrogen in limited nutrient conditions and in the use of urea as a nitrogen source. In this study, the Ni levels were almost 10-fold more on bricks with a heavy infestation of the cyanobacteria in comparison to the brick with no algae. On the contrary, the Co levels were almost 15 times more in the bricks with a heavy infestation of algae than with minimal or no alga infestations. Thus, the presence of high levels of metals probably contributed to the high prevalence of algae infestations of the pavement bricks.

## Conclusion

The slippery pavement is hazardous to people walking on it and may result in injury. Thus, the study showed that slippery bricks had high levels of metals in comparison to non-slippery bricks. It was found that bricks with heavy metal contents such as Al, V, Fe, Ti, Mn, Pb, Zn, Ni and Co had high concentrations of algae, whilst bricks with low metal content had low presence of algae. The other factors that promote the growth of algae on the brick pavements are identified as alkaline pH, high nutrients (nitrates and phosphates), high solar radiation, high humidity and low EC.

### Recommendation

To avoid the algae from developing on bricks (i.e. to reduce hazards from slippery pavement), it is recommended that, firstly, brick manufacturers should reduce the metal contents of Zn, Mn, Fe, Ti, Ni, Pb, Al and V. Secondly, there is a need for further research on metal composition of bricks so as to reduce the proliferation of these microorganisms on bricks during rain periods.
